# Lack of evidence for expression and function of IL-39 in human immune cells

**DOI:** 10.1371/journal.pone.0242329

**Published:** 2020-12-01

**Authors:** Florence Ecoeur, Jessica Weiss, Simone Schleeger, Christine Guntermann

**Affiliations:** Autoimmunity, Transplantation and Inflammation, Novartis Institutes for BioMedical Research, Basel, Switzerland; Universite de Nantes, FRANCE

## Abstract

Members of the IL-6/IL-12 cytokine family are critical regulators of innate and adaptive immunity and have emerged as key players controlling inflammatory and autoimmune disorders. This cytokine family comprises of IL-12, IL-23, IL-27, and IL-35, each consisting of distinct α- and β-cytokine subunits that form heterodimers. A new member of this family, IL-39, was identified in the murine species and was shown to consist of the IL-23p19 and Epstein-Barr Virus-induced 3 (EBI3) subunits. Subsequently, it was shown that IL-39 was implicated in the immunopathogenesis of murine experimental lupus erythematosus. The existence of IL-39 in the human system has yet to be confirmed. Based on the clinical success of IL-23p19 neutralizing approaches in moderate-to-severe psoriasis, anti-IL-23p19 antibodies in the clinic may not only neutralize IL-23, but additionally IL-39, implying that IL-39 might also contribute to the pathogenesis of psoriasis. It is therefore pivotal to demonstrate IL-39 expression and to characterize its function in the human system. In this study, we provided evidence for the existence of secreted heterodimeric p19 and EBI3 complexes in supernatants originating from p19 and EBI3 transfected HEK293FT cells. We attempted to detect IL-39 expression from stimulated human primary B cells, human keratinocytes and in *vitro* polarized human macrophages. Whereas, the expression of *p19* and *EBI3* mRNA was elevated, we failed to detect p19 and EBI3 heterodimers. Functional assays were conducted with conditioned media containing human IL-39 or with a human recombinant IL-39 Fc protein. Immune cells targeted by IL-39 in mouse, such as neutrophils and PBMCs, did not respond to human IL-39 stimulation and IL-39 failed to activate STAT3 in a reporter cell line. These results suggest that, while the secretion of p19/EBI3 complexes can be forced in human cells, it is secreted below the lower quantity of detection or it has no functional role.

## Introduction

Members of the IL-6/IL-12 cytokine family consist of IL-12, IL-23, IL-27 and IL-35 and have unique structure properties in that they form heterodimers comprising distinct α- and β-subunits. Members of this family have emerged as critical players in promoting and suppressing many immune responses under physiological and pathological conditions, mainly by influencing the developmental fates of naive T and B cells [1, 2]. Pro-inflammatory cytokines of this family include IL-12 and IL-23 that promote Th1 and Th17 differentiation, respectively, and these cells have been implicated in the etiopathology of various autoimmune diseases including psoriasis, rheumatoid arthritis, psoriatic arthritis and multiple sclerosis [3–6]. By contrast, IL-27 and IL-35 have rather protective and immunosuppressive functions, limiting excessive inflammation by inducing IL-10 producing regulatory T-cells and by expanding regulatory B cells, respectively [7–10]. A new member of the IL-6/IL-12 family, termed IL-39, was recently discovered in mice. It is composed of a heterodimeric complex consisting of the p19 α (shared with IL-23) and of the EBI3 β (shared with IL-27/IL-35) subunits [11]. IL-39 is produced *in vivo* and *in vitro* by activated mouse B cells that mediate inflammation in lupus-prone mice [11]. IL-39 induced the differentiation and/or expansion of neutrophils and IL-39-activated neutrophils increased IL-39 expression in B cells by secreting B-cell activating factor (BAFF) [12]. IL-39 binds to the IL-23 receptor/Gp130 receptor complex and activates STAT1 and STAT3 transcription factors in murine B cells and neutrophils [11, 12]. There is evidence that IL-39 is associated with lupus immunopathogenicity in mice because shRNA-mediated knockdown of the p19 or EBI3 subunits in GL7^+^ B cells followed by adoptive transfer attenuated inflammation in lupus-like mice [11]. Furthermore, a polyclonal anti-IL-39 antibody ameliorated lupus-like pathology in mice confirming that IL-39 is pro-inflammatory and could therefore represent a potential therapeutic target for the treatment of autoimmune diseases, such as systemic lupus erythematosus [13]. There is a paucity of data on IL-39 in the human system and expression of this cytokine in man has yet to be confirmed. A report showed that in TLR3 activated human keratinocytes *p19* and *EBI3* gene expression was upregulated, suggesting a possible cellular source of human IL-39 [14]. A recent publication assessing the combinatorial potential of chain pairing in the IL-12 family failed to detect secreted IL-39, whereas other cytokines of the IL-6/IL-12 family were detected [15]. Clinically, several antibodies against IL-23 p19 show good efficacy in moderate-to-severe psoriasis and psoriatic arthritis [4, 16] and it remains to be established whether IL-39 contributes to disease pathogenesis. It could be that the impressive efficacy of anti-p19 blocking antibodies in the clinic is due to neutralization of both cytokines, IL-23 and IL-39. Because of the potential pro-inflammatory and pathogenic property of IL-39, we attempted to clarify whether IL-39 exists and whether it has a biological function in the human system.

When the two subunits were overexpressed, we were able to demonstrate the existence of IL-39 as a secreted cytokine. Potential cellular sources such as activated B cells, macrophages and keratinocytes expressed the *p19* and *EBI3* mRNA, however, we were unable to detect the p19 and EBI3 protein complexes produced by these cells. In addition, we conducted functional assays using a disulfide-linked human IL-39 chimeric protein or conditioned media containing IL-39. Immune cells, including neutrophils and PBMCs, that were previously shown to respond to IL-39 were not activated by the cytokine. Consistent with this, IL-39 Fc or concentrated supernatants containing the p19/EBI3 heterodimer did not induce STAT3 activation in a reporter cell line, whereas IL-6, the positive control, did. Finally, using an IL-23-dependent reporter cell line, we further demonstrated that IL-39 did not block IL-23 receptor function and did not interfere with IL-23-induced STAT3 activation. Our results suggest that IL-39 secreted by immune cells is too low to be detectable or alternatively, its existence and function in the human system is doubtful.

## Materials and methods

### Human study approval

Anonymized buffy coats from healthy volunteers were collected through the InterRegionale Blutspende of the Swiss Red Cross in Bern, Switzerland. Blood from healthy volunteers was provided under informed consent and collected through the Novartis Tissue Donor Program (TRI0128) in accordance with the Swiss Human Research Act and approval of the responsible ethic committee (Ethikkommission Nordwest- und Zentralschweiz number: 329/13).

### Human cells

HEK293FT cells (ThermoFisher Scientific) were cultured in DMEM high glucose (4.5 g/L) and pyruvate (Gibco), supplemented with 10% Fetal Bovine Serum Gold (GE Healthcare), 1% Non-Essential Amino Acids (Gibco) and 1% Penicillin-Streptomycin. The STAT3 reporter cell line, HEK-Blue^TM^ IL-6 and the IL-23 responsive STAT3 reporter cell line, HEK-Blue^TM^ IL-23 were purchased from Invivogen. The cells were cultured in DMEM high glucose (4.5 g/L) and Glutamax (Gibco), supplemented with 10% Fetal Bovine Serum Gold (GE Healthcare), 1% Penicillin-Streptomycin (Gibco), 100 μg/ml Normocin (Invivogen) and 1X HEK-Blue selection (Invivogen).

### Plasmids

The p19, EBI3 and EBI3-His plasmids were kindly provided by Dr Simone Popp (Novartis, Basel, Switzerland). The human IL-23 p19 (aa20-189), EBI3 (aa21-229) or C-terminally 10 x His-tagged EBI3 ORFs were inserted into the mammalian expression vector pRS5a. The IL-23 receptor plasmid was purchased from OriGene, the corresponding untagged ORF (NM_144701) being inserted into the pCMV6-XL5 expression vector.

### HEK293FT cell transfection with IL-23 p19/EBI3 plasmids

Four millions cells were seeded in a 10 cm petri dish (Corning) and incubated overnight at 37°C with CO_2_. The following plasmids were used for Lipofectamine-mediated cell transfection: pRS5a-IL23p19 (2 μg per petri dish), pRS5a-EBI3 or pRS5a-EBI3-His10 (3 μg per petri dish). Two days after cell transfection, the supernatants were collected and concentrated with a Centriprep YM-30 tube (Millipore) by a 30-minute centrifugation step at 3000 g and 4°C. The concentrated eluates were subsequently used in co-immunoprecipitations and ELISA studies.

### Intracellular staining and flow cytometry

HEK293 FT cells that were either mock transfected or transfected with p19/EBI3-His plasmids were washed, fixed and permeabilized in Cytofix/Cytoperm (BD Biosciences) according to the manufacturer`s instructions prior to intracellular staining with AX488 labelled p19 antibody (Thermofisher Scientific) and with APC labelled EBI3 antibody (R&D Systems). For staining of LPS/BAFF activated B cells, cells were incubated for 4 hrs at 37°C with 5 ng/ml of PMA (Sigma) and 500 ng/ml of Ionomycin (Calbiochem) in the presence of 10 μg/ml Brefeldin A (Sigma). B cells were washed, fixed and permeabilized prior to intracellular staining with AX488 labelled p19 antibody (ThermoFisher Scientific) and/or with APC labelled EBI3 antibody (R&D Systems). Data were acquired on a LSR Fortessa (BD Bioscience) and analyzed using FlowJo software (Tree Star).

### IL-23 p19/EBI3 co-immunoprecipitation

Concentrated supernatants originating from p19 and EBI3 or EBI3-His transfected HEK293FT cells were pre-cleared with Protein G sepharose beads (GE Healthcare) for 30 minutes at 4°C on a rotating wheel. The pre-cleared supernatants were diluted with PBS-0.05% Tween 20 buffer containing protease inhibitors (Roche) and incubated with 3μg of anti-p19 (Invitrogen Thermo Fisher Scientific) or anti-His (Abcam) antibodies for 3 hrs at 4°C on a rotating wheel. Protein G sepharose beads were then added to the samples and incubated for 1 hr, followed by washing, addition of 2 x sample buffer (Biorad) and boiling. Proteins were separated by SDS-PAGE, transferred to PVDF membranes and immunoblotted overnight with p19 (Invitrogen), 6xHis-Tag (Abcam), EBI3 (Cloud-Clone-Corp) or EBI3-biotin (Biorbyt) antibodies. Protein bands were visualized by enhanced chemiluminescence (Western bright Quantum, Advansta).

### IL-39 ELISA for detection of the p19/EBI3 heterodimer and to determine selectivity against related IL-23 or IL-27 cytokines

96-well plates (Nunc Maxisorp) were coated with an antibody against p19 (R&D Systems, 0.7 μg/ml) overnight at 4°C, washed and blocked with PBS 1% BSA. Concentrated supernatants from p19 and/or EBI3-His transfected HEK293FT cells or titrations of a human IL-39 IgG1-Fc fusion protein or IgG1-Fc control protein (R&D Systems) were incubated for 2 hrs at room temperature. After washing of the plates, biotinylated EBI3 antibody (Biorbyt, 3 μg/ml) was added for 1 hr, followed by washing and by incubation with HRP-conjugated Avidin (Biolegend). In order to determine whether the IL-39 ELISA recognizes other p19- or EBI3-containing cytokines, 200 nM recombinant IL-23 or IL-27 cytokines (R&D Systems) were added to the anti-p19 coated and blocked plates. After 2 hrs of incubation at room temperature, the plates were washed and biotinylated EBI3 antibody was added for 1 hr. The plates were washed and incubated with HRP-conjugated Avidin (Biolegend). After further washing, the reaction was visualized with TMB substrate (eBiosciences) and stopped with sulfuric acid before absorbance was measured at 450 nm. The sensitive human IL-23 MAX^TM^ ELISA was purchased from Biolegend and p19 and/or EBI3-containing supernatants from transfected HEK293 FT cells were subjected to according to the manufacturer`s recommendations.

### Isolation and stimulation of human B cells, monocytes or neutrophils

Human B cells were purified from PBMCs by immunomagnetic separation using B-cell isolation kits according to the manufacturer`s recommendation (Stem Cell Technologies). B cells were stimulated (1 x 10^6^ B cells/well) for 48 hrs with LPS (1 μg/ml, Sigma) and/or with BAFF (50 ng/ml, Cedarlane). Cells were either analyzed by quantitative RT-PCR to quantify *p19* and *EBI3* gene expression or were prepared for intracellular FACS analysis to detect p19/EBI3 protein expression. Human monocytes were obtained from PBMCs after immunomagnetic isolation according to manufacturer’s instructions (Stem Cell Technologies) and cultivated for 6 days with 40 ng/mL M-CSF (R&D Systems). Afterwards, they were differentiated for 48 hrs in M0, M1 or M2 macrophages with either M-CSF alone or M-CSF plus IFNγ (50 ng/ml, R&D Systems) or M-CSF plus IL-4 (50 ng/ml, R&D Systems) respectively. The cells were finally stimulated for 24 hrs with 1 ng/ml LPS (Sigma-Aldrich) followed by supernatant collection and cell pellet lysis. The supernatants were subjected to p19-EBI3 ELISA and the cells lysates were used to quantify *p19* and *EBI3* gene expression. Human neutrophils were directly isolated from Na-Heparin collected fresh blood by immunomagnetic separation according to the manufacturer’s recommendations (Stem Cell Technologies). Neutrophils (1 x 10^5^ cells/well) were incubated for 24 hrs with concentrated supernatants from p19 and/or EBI3-His transfected HEK293FT cells or 1μg/ml of human IL-39 IgG1-Fc fusion protein or IgG1-Fc control protein (R&D Systems). Cells were then analyzed by quantitative RT-PCR to assess *BAFF*, *TNFα*, *IL6* and *IL8* gene expression.

### Stimulation of HaCaT cells to induce IL-23 p19/EBI3 expression

The HaCaT keratinocyte cell line was purchased from AddexBio and cultured in RPMI 1640 medium supplemented with Glutamax, Penicillin/Streptomycin (all from Gibco) and 10% FCS (PAA). HaCaT cells were incubated in a 96-well microtiter plate (1 x 10^4^ cells/well) and stimulated with the TLR3 agonist Poly IC (30 μg/ml, Invivogen). After 48 hrs, supernatants were collected and subjected to a p19/EBI3 ELISA, cells were harvested and were further processed for quantitative RT-PCR analysis.

### Stimulation of human PBMCs with IL-39-chimeric Fc protein

PBMCs were obtained from human buffy coats by density gradient centrifugation using Ficoll-Paque. The cells (5 x 10^5^ PBMCs/well) were stimulated with antibodies against CD3 (1 μg/ml, clone OKT-3, BioXcell) and CD28 (1 μg/ml, clone CD28.2, BioLegend) in the presence of human IL-39 IgG1-Fc fusion or IgG1-Fc control protein (3 μg/ml). Supernatants were collected and IFNγ or IL-17A cytokines were quantified by ELISA (eBioscience) after 48 hrs or 72 hrs of incubation, respectively.

### Stimulation of HEK-Blue^TM^ IL-6 cells to detect IL-39 dependent STAT3 activity

HEK-Blue^TM^ IL-6 cells are stably transfected with the human IL-6R gene and a STAT3-inducible secreted embryonic alkaline phosphatase (SEAP) reporter gene. Briefly, 4 x 10^6^ cells were transfected using lipofectamine with 16 μg pCMV6-XL5-IL-23 receptor plasmid (OriGene). After 48 hrs incubation, cells were stimulated for 20 hrs with concentrated supernatants from p19 and/or EBI3-His transfected HEK293FT cells or 10 μg/ml of human IL-39 IgG1-Fc fusion protein or IgG1-Fc control protein or 1 ng/ml IL-6 (BioLegend) as positive control. The supernatants were then collected and subjected to SEAP determination using QUANTI-Blue^TM^ according to manufacturer’s instructions (Invivogen). A transfection efficiency control was performed before cell stimulation by lysing cell pellets to confirm *Gp130* and *IL6R* gene expression.

### Stimulation of HEK-Blue^TM^ cells with IL-23 in the presence of IL-39 IgG1-Fc fusion protein

In order to determine whether IL-39 would interfere with IL-23-induced signaling responses, an IL-23 responsive reporter cell line was purchased (Invivogen). The HEK-Blue^TM^ IL-23 cells are stably transfected with the human IL-23R gene and a STAT3-inducible secreted embryonic alkaline phosphatase (SEAP) reporter gene. Cells (5 x 10^4^/well) were stimulated for 20 hrs with various concentrations of recombinant human IL-23 either in the presence or absence of various concentrations of IL-39 IgG1-Fc fusion protein or IgG1-Fc control protein (2–200 pM). The supernatants were then collected and subjected to SEAP determination using QUANTI-Blue^TM^ according to manufacturer’s instructions (Invivogen).

### RNA extraction and quantitative RT-PCR

Total RNA from human cells was extracted using RNeasy kit including a DNAse I digestion step according to the manufacturer`s protocol (Qiagen). cDNAs were prepared with the High Capacity cDNA Reverse Transcription Kit (Applied Biosystems). Quantitative RT-PCR analysis was performed using a TaqManViiA7 machine (Applied Biosystems). The expression level of each gene was normalized to β-glucoronidase (*Gus*) expression (Applied Biosystems, 431088E) using the ΔΔCt method. The following probes were used for quantitative RT-PCR: *p19* (Hs00372324_m1), *EBI3* (Hs01057148_m1), *BAFF* (Hs00198106_m1), *TNFa* (Hs99999043_m1), *IL6* (Hs00985639_m1), *IL8* (Hs00174103_m1), *IL23R* (Hs00332759_m1) and *Gp130* (Hs00174360_m1).

## Results

### Human IL-39 exists as a secreted IL-23p19/EBI3 heterodimeric cytokine

**Fig 1:**
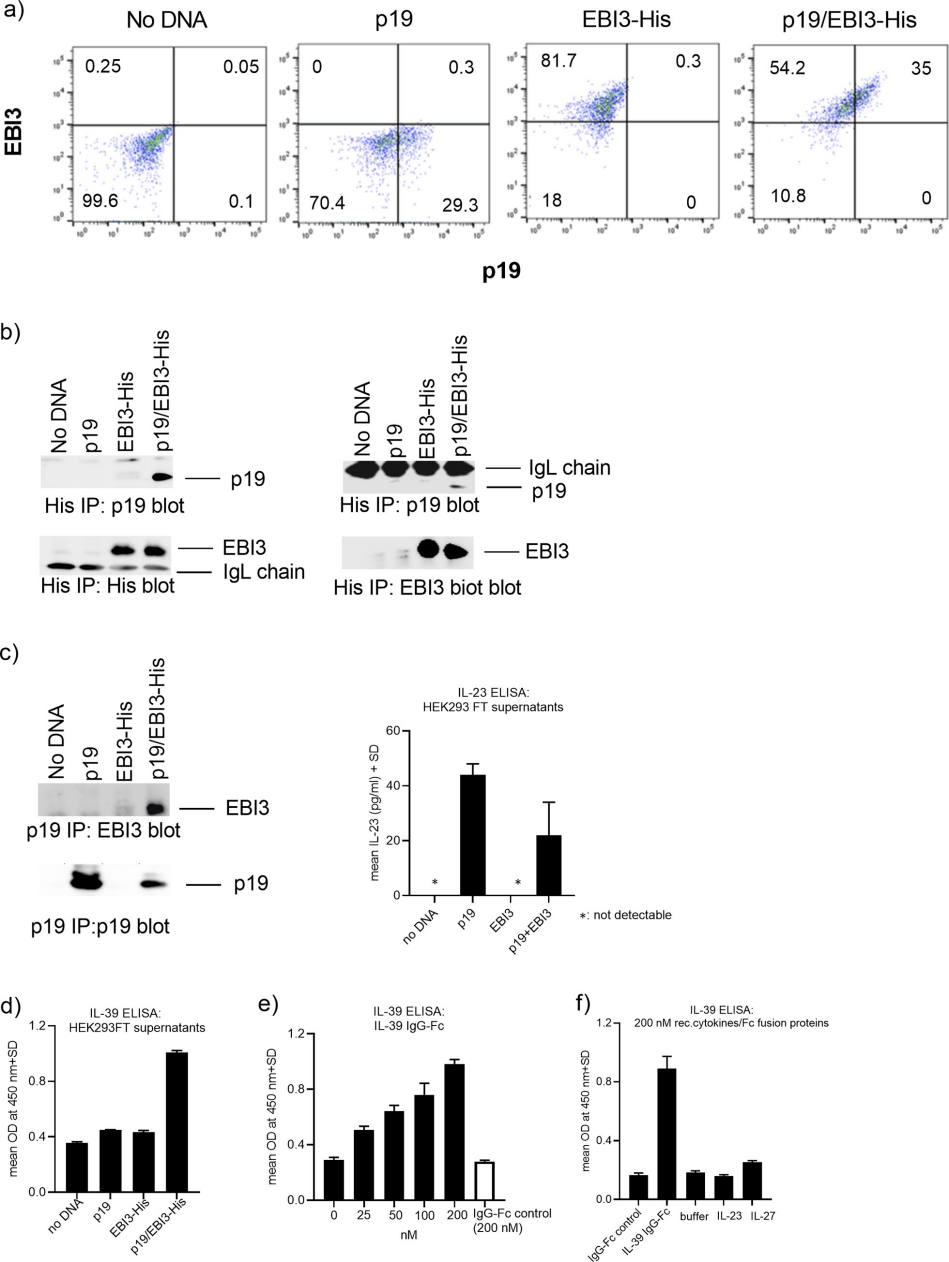
IL-23p19 and EBI3 proteins interact and form a stable, secreted heterodimeric complex. (A) Co-expression of p19 and EBI3 proteins at the intracellular level. Intracellular FACS staining of HEK293FT cells that were transiently transfected with p19 or with His-tagged EBI3 plasmids alone or co-transfected with p19 and His-tagged EBI3 plasmids. Cells were fixed and permeabilized, followed by intracellular staining using p19 and EBI3-specific antibodies. (B and C) Co-immunoprecipitation studies using pre-cleared supernatants originating from tagged or untagged p19/EBI3 double transfected HEK293FT cells. Samples were subjected to anti-His Ab (B) or anti-p19 antibody (C) immunoprecipitation, followed by anti-His, p19 and EBI3 Western blotting. In addition, supernatants from transfected HEK293 FT cells were monitored for the presence of the IL-23 cytokine using a sensitive IL-23 ELISA. Representative results from at least three independent experiments are shown. (D and E) Detection of p19/EBI3-His heterodimeric complex by ELISA. Microtiter plates were coated with an anti-p19 Ab, followed by blocking. The wells were either incubated with p19/EBI3-His containing supernatants (D) or with human IL-39 IgG1-Fc fusion or IgG1-Fc control protein (E). After washing, the wells were incubated with biotinylated EBI3 Ab, followed by washing and by incubation with HRP-conjugated Avidin. (F) Specificity of IL-39 ELISA against the related IL-23 or IL-27 cytokines. Microtiter plates were coated with an anti-p19 Ab, blocked and incubated with recombinant human IL-23 or IL-27 cytokines or with the IL-39 IgG1-Fc fusion protein. After washing, the wells were incubated with biotinylated EBI3 Ab, followed by washing and by incubation with HRP-conjugated Avidin. Representative results from at least three independent experiments are shown.

In order to test whether the human p19/EBI3 heterodimeric complex can form, we transiently co-transfected HEK293FT cells with plasmid constructs encoding for the two subunits. After 48 hrs of transfection, HEK293FT cells were used for intracellular staining followed by FACS analysis to detect cells that co-expressed the two subunits. We found that a substantial proportion of HEK293FT cells were efficiently transfected with the p19 or EBI3 plasmid alone or in combination (Fig 1A). We further investigated whether the p19/EBI3 heterodimeric complex can be detected. We used the concentrated supernatants derived from the co-transfected cells and performed immunoprecipitation studies to demonstrate that the p19 and EBI3 subunits formed a secreted heterodimer. When the supernatants were pre-cleared with Protein G sepharose beads and His-tagged EBI3 proteins were immunoprecipitated using an anti-His Ab, followed by p19 western blotting, a protein band migrating at 19 kDa was detectable in the His-EBI3 immunoprecipitates (Fig 1B). As a specificity control, single transfections with either p19 or the EBI3-His plasmids did not reveal p19 in the EBI3-His immunoprecipitations. We corroborated these results by performing reciprocal p19 co-immunoprecipitations using supernatants from untagged p19 and EBI3 transfected HEK293FT cells (Fig 1C). These results demonstrate that p19 and EBI3 can form a stable complex that can be secreted by the cells. A surprising finding from our studies was that we consistently observed that the p19 subunit could be detected in the conditioned medium even when the HEK293FT cells were transfected with the p19 plasmid alone. It is well known in the field that the human p19 subunit could only be detected in supernatants when it is complexed with IL-23p40 to form IL-23 [17]. We speculated that the transfected p19 subunit could pair intracellularly with endogenous p40 to form IL-23 which was then secreted by the cells. In order to establish whether the cells secreted IL-23, we subjected the supernatants to a commercially available, sensitive IL-23 ELISA and we found that samples that were either transfected with the p19 plasmid only or with the p19/EBI3-His plasmids clearly contained detectable levels of the IL-23 cytokine (Fig 1C). As expected, we were unable to detect secreted IL-23 in samples originating from untransfected or EBI3-His transfected cells (Fig 1C).

In the next set of experiments, we confirmed the expression of the secreted p19/EBI3-His heterodimer by ELISA when p19 present in the supernatants was captured by p19 specific Abs and the associated EBI3-His was detected by anti-EBI3 Abs (Fig 1D). As background controls, supernatants originating from either mock or single p19 or EBI3-His transfected HEK293FT cells were used in the ELISA. We consistently observed an approximately 2.5 fold increase in the optical density readings when the double transfected supernatants were used in the ELISAs. Using the same ELISA set-up, a commercially available disulfide-linked IL-39 IgG1-Fc fusion protein was used. The IL-39 fusion protein concentration-dependently increased the optical density readings in the ELISA, whereas an IgG1-Fc control protein gave only background values (Fig 1E). In order to confirm that our ELISA is specific for IL-39 and does not react with related cytokines containing the p19 or EBI3 subunits we used recombinant IL-23 (p19/p40) or IL-27 (p28/EBI3) cytokines in this ELISA set-up. As expected, only the IL-39 IgG1-Fc fusion protein clearly reacted in the IL-39 ELISA, whereas the IgG1-Fc control protein, the IL-23 and IL-27 cytokines did not induce any significant signal above background, indicating the validity and the specificity of the IL-39 ELISA assay (Fig 1F). In summary, employing immunoprecipitation and ELISA techniques we were able to detect IL-39 cytokine in supernatants originating from transfected HEK293FT cells.

### Expression of *p19/EBI3* in human B cells and in keratinocytes

**Fig 2:**
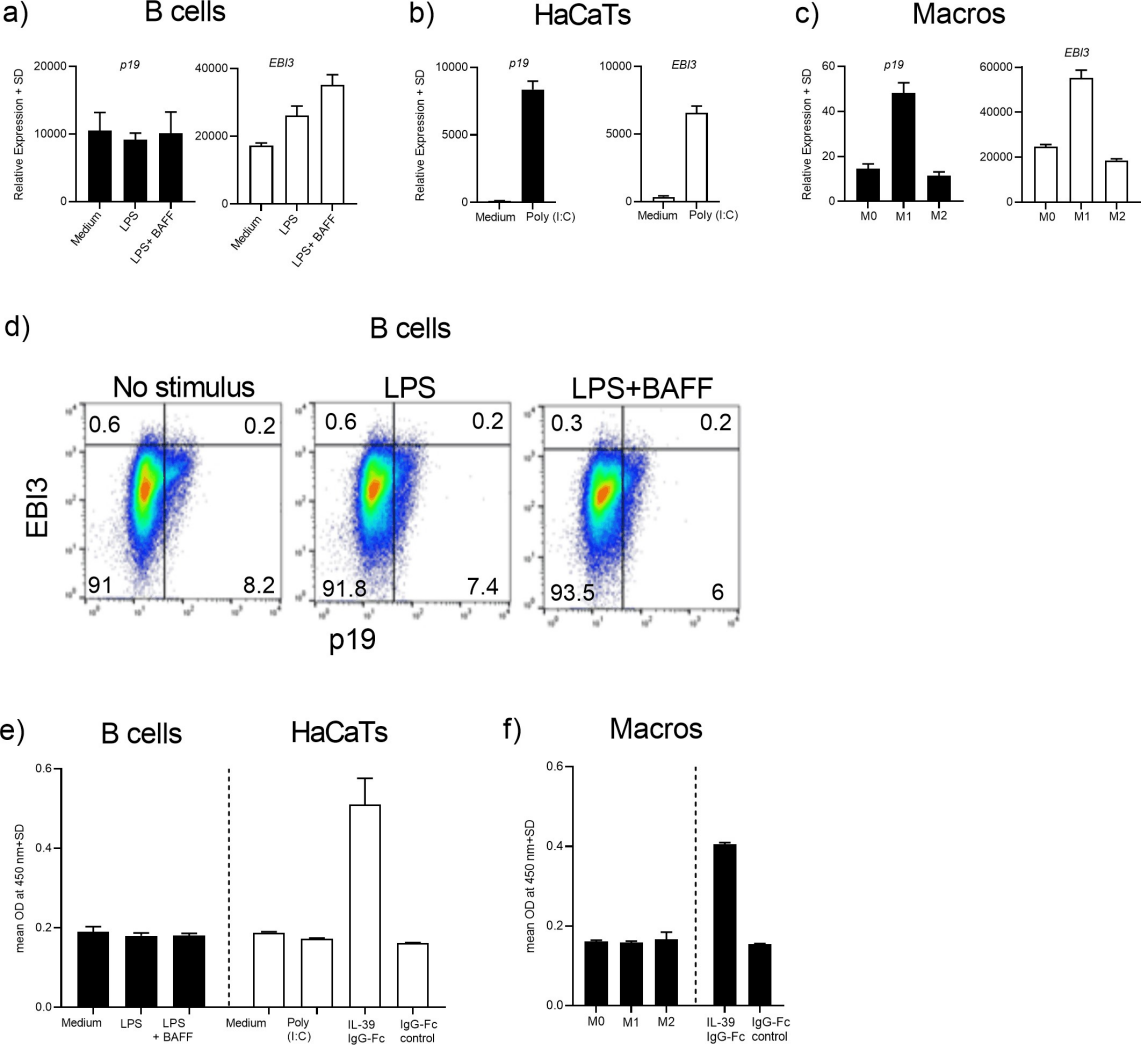
mRNA expression, but no protein expression of the IL-39 subunits from human immune cells and keratinocytes. (A) Purified human B cells were incubated with medium only or stimulated with LPS (1 μg/ml) or with a combination of LPS (1 μg/ml) and BAFF 50 ng/ml for 48 hrs. (B) HaCaT cells were incubated with medium or were stimulated with the TLR3 agonist Poly IC (30 μg/ml) for 48 hrs. (C) Polarized M0, M1 or M2 macrophages were subjected to quantitative RT-PCR analysis. (A-C) mRNA was extracted and transcript levels were measured by quantitative RT-PCR. Gene expression was normalized to β-glucoronidase levels and expressed as arbitrary units. (D) Intracellular FACS staining of B cells from (A). (E and F) Supernatants originating from cultures (A-C) or an IL-39 IgG1-Fc fusion protein were used in ELISAs where anti-p19 Ab was used for capturing of p19 and anti-EBI3 served as the secondary Ab. Graphs are representative of two independent experiments.

After our observation that human IL-39 was secreted by transfected HEK293FT cells, we attempted to identify the cellular sources of the p19 and EBI3 heterodimer in human cells. In the mouse system, it was reported that IL-39 was secreted by B cells following *in vitro* stimulation with LPS [11]. IL-39 secreted by activated B cells induced differentiation and/or expansion of neutrophils in lupus-prone mice [12]. Activated neutrophils responded by producing BAFF which exerted a positive feedback loop on B cells leading to enhanced IL-39 expression [12]. In another publication, it was demonstrated that human primary keratinocytes and the HaCaT keratinocyte cell line respond to TLR3 ligation by upregulating *p19* and *EBI3* gene expression [14]. Based on these published results, we assessed whether human B cells or HaCaT cells could be a cellular source for the p19 and EBI3 complex. Human B cells were purified from buffy coats and were stimulated for 48 hrs with LPS alone or with a combination of LPS and BAFF. Gene expression of *p19* and *EBI3* was determined by quantitative RT-PCR analysis. Both genes were constitutively expressed in B cells and, in the case of *EBI3*, expression was further upregulated after LPS or LPS plus BAFF activation (Fig 2A). Next, we stimulated HaCaT cells with the TLR3 agonist Poly IC for 48 hrs and we found that *p19* and *EBI3* expression levels were significantly upregulated following stimulation (Fig 2B). Because macrophages and DCs constitute the main cellular source for the pro-inflammatory IL-23 (p19/p40) cytokine, we also determined if the p19 and EBI3 subunits were expressed in macrophages that had been polarized towards the M0, M1 or M2 phenotype. Consistent with the finding that IL-23 represents a marker of M1 polarized macrophages, we also observed elevated expression of *p19* and *EBI3* primarily in M1 polarized macrophages (Fig 2C). In the next set of experiments, it was investigated whether the human p19 and EBI3 heterodimer was expressed at the protein level by using FACS analysis or ELISA technique. LPS- or LPS/BAFF-stimulated B cells were stained with p19 and EBI3-specific antibodies and were prepared for intracellular p19/EBI3 FACS analysis. We were unable to detect co-expression of p19 and EBI3 at the intracellular level, irrespective of the stimulation regime (Fig 2D). In fact, none of the proteins were up-regulated after B-cell stimulation. The culture supernatants from the activated B cells, HaCaT cells and polarized macrophages were used in ELISAs where p19 was captured by the primary antibody and the EBI3 in the complex was revealed by the secondary antibody. The positive control for IL-39 in the ELISA set-up was the IL-39 IgG1-Fc fusion protein. Whereas, we observed an increased signal in the ELISAs when the IL-39 chimeric protein was used, we were unable to detect the presence of the p19/EBI3 heterodimeric cytokine complex in the supernatants originating from activated B cells, Poly IC-stimulated HaCaT cells or from polarized macrophages (Fig 2E and 2F). In summary, whereas *p19* and *EBI3* gene expression was readily identified in putative cellular sources, the IL-39 protein was not detectable.

### IL-39 does not elicit functional responses in human immune cells

**Fig 3:**
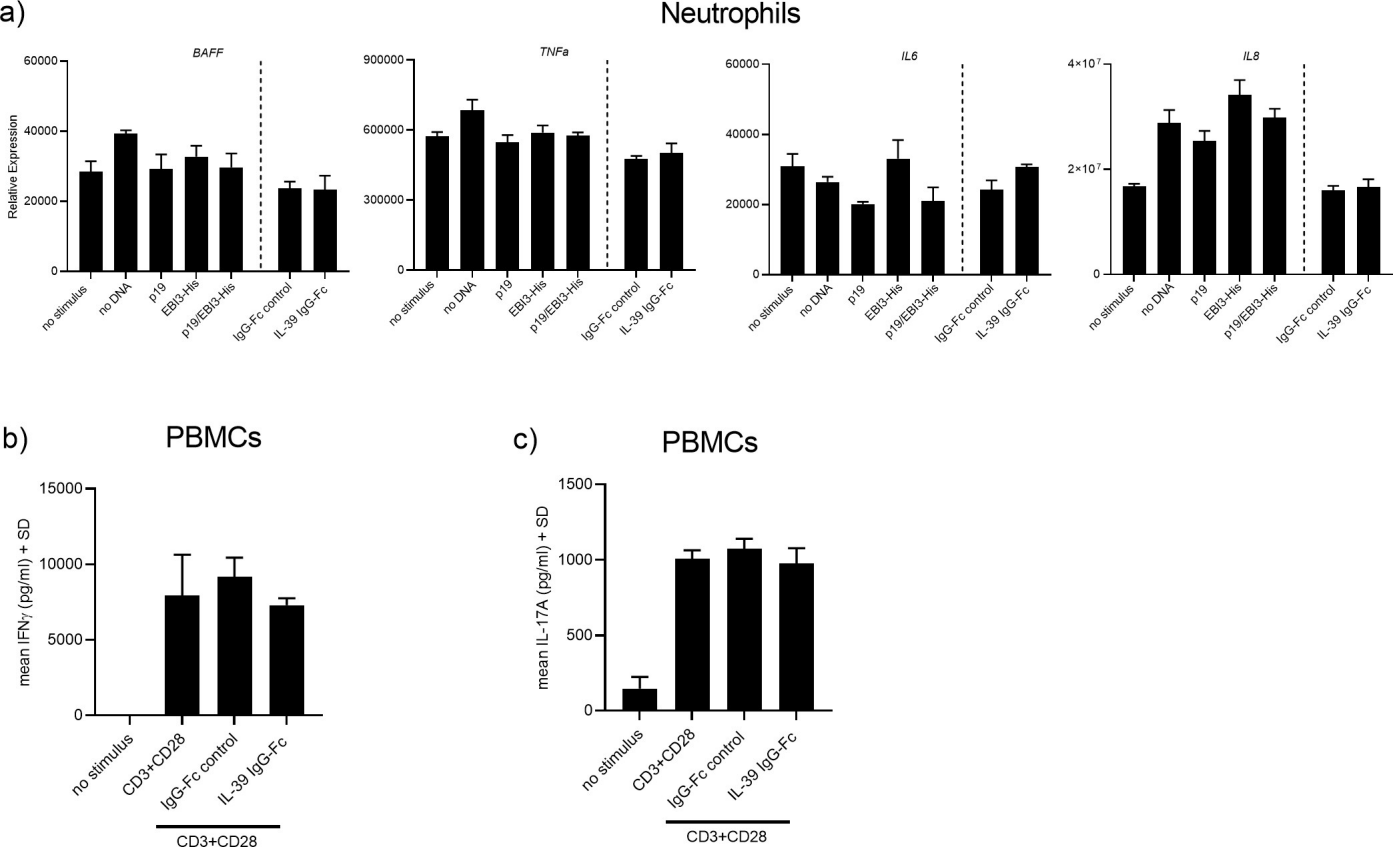
Human IL-39 does not activate immune cells and does not activate STAT3. (A) Human neutrophils were incubated with IL-39 containing concentrated supernatants or with recombinant human IL-39 fusion or IgG1-Fc control protein (both at 1 μg/ml). After 24 hrs, the reported IL-39-controlled target *BAFF* and other neutrophil mediator genes were quantified by quantitative RT-PCR. Gene expression was normalized to β-glucoronidase levels and expressed as arbitrary units. Graphs are representative from two experiments containing three technical replicates each. Error bars represent the SD. (B and C) PBMCs were stimulated with CD3+CD28 Abs in the presence of IL-39 IgG1-Fc fusion or IgG1-Fc control protein (3 μg/ml). After 72 hrs of incubation, IFNγ and IL-17A concentrations present in the supernatants were determined by ELISAs. Data represent triplicate measurements of three independent experiments.

Previous studies in the murine system identified neutrophils as cellular targets for IL-39 [12]. The mechanism how IL-39 is thought to induce intracellular signaling is via binding to the cognate IL-23 receptor and Gp130 subunits resulting in activation of STAT1 and STAT3 [11, 18]. We further explored whether the IL-39 IgG-Fc fusion protein or concentrated supernatants originating from p19/EBI3-His co-transfected HEK293FT cells that express the p19/EBI3 heterodimeric complex could trigger functional and signaling responses. Purified human neutrophils were exposed for 24 hrs with concentrated HEK293FT cell supernatants containing either the single p19 or EBI3-His subunits or the p19/EBI3-His heterodimeric protein complex. Compared to supernatants from control or single transfected HEK293FT cells, there were no changes in *BAFF*, *TNFα*, *IL6* or *IL8* mRNA expression in neutrophils stimulated with the p19/EBI3-His containing supernatants (Fig 3A). Similar results were obtained when neutrophils were stimulated with IL-39 chimeric protein (1 μg/ml). There was no induction by the IL-39 protein *versus* IgG1-Fc control protein (Fig 3A). Addition of the human IL-39 fusion protein has reported biological activity on murine splenocytes and the IL-39 chimeric protein was shown to potently induce IFNγ production with an EC_50_ activity of 50–250 ng/ml (R&D systems). Using human PBMCs, we only could detect significant amounts of IFNγ in the culture supernatants when the cells were stimulated with CD3+CD28 Abs plus IL-39 chimeric protein for 72 hrs. However, incubation of IL-39 chimeric protein to the co-stimulated PBMCs did not further enhance IFNγ secretion (Fig 3B), even when used at a 12 to 60 fold higher concentration than the reported EC_50_ value (3 μg/ml final concentration). Because IL-23 shares the p19 subunit with IL-39, stimulates IL-17A production from T-cells, we also investigated whether IL-39 would affect IL-17A secretion induced by CD3+CD28 Abs. There was no effect of recombinant IL-39 chimeric protein on IL-17A secretion produced by human PBMCs (Fig 3C).

### IL-39 fails to induce STAT3 activity and does not interfere with IL-23-mediated signaling

**Fig 4:**
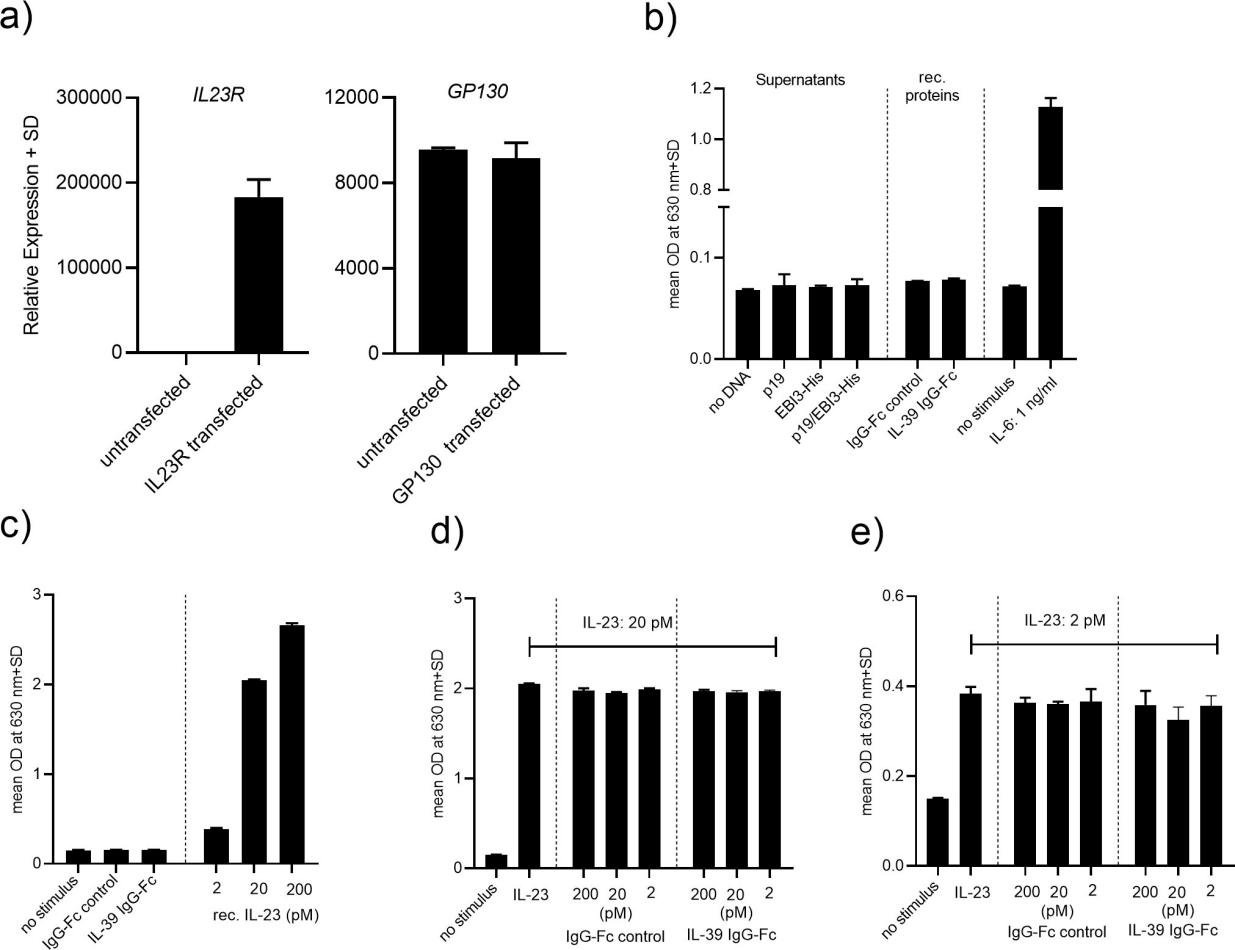
Human IL-39 does not activate STAT3 and does not inhibit IL-23-induced signaling. (A) HEK-Blue^TM^ STAT3 IL-6 cytokine reporter cells were transiently transfected with a plasmid encoding for the human IL-23 receptor and after 48 hrs, expression of the IL-39 receptor genes *IL23r* and *Gp130* were quantified by quantitative RT-PCR. The Ct values for *IL23r* and *Gp130* were 22 and 26, respectively. (B) HEK-Blue^TM^ STAT3 transfected cells from (A) were incubated with recombinant, human IL-6 (1 ng/ml), with IL-39 IgG1-Fc fusion or IgG1-Fc control protein (10 μg/ml) or with concentrated IL-39 containing supernatants originating from HEK293FT cells transfected with either p19, EBI3-His or with a combination of p19/EBI3-His. After 20 hrs, secretion of the STAT3-induced SEAP reporter was measured by QUANTI-Blue^TM^. (C and D) IL-39 does not antagonize IL-23-mediated signal transduction. HEK-Blue^TM^ IL-23 cells stably expressing the human IL-23 receptor and a STAT3-inducible SEAP reporter were stimulated for 20 hrs with various concentrations of recombinant human IL-23 to induce the STAT3-mediated SEAP reporter. Alternatively, the cells were incubated with a cocktail containing IL-23 plus different concentrations of IL-39 IgG1-Fc fusion or IL-23 plus IgG1-Fc control protein. Secretion of the STAT3-induced SEAP reporter was measured by QUANTI-Blue^TM^.

In the next set of experiments, we assessed whether IL-39 could trigger signaling responses downstream of the IL-39 receptor (IL-23r/Gp130). We chose HEK293 cytokine reporter cells that stably express the IL-6 receptor, the signal transducing subunit Gp130 and a STAT3-inducible SEAP reporter gene (HEK-Blue^TM^ IL-6). These cells respond to IL-6 stimulation by activating the STAT3 pathway resulting in enhanced SEAP reporter gene activity and SEAP secretion. We transiently transfected these reporter cells with a plasmid encoding the human IL-23 receptor, 48 hrs after transfection, the cells were stimulated with IL-39 chimeric protein or with supernatants from HEK293FT transfected cells containing p19, EBI3-His or the heterodimeric p19/EBI3-His complex. As a positive control for STAT3 pathway activation, cells were stimulated with recombinant human IL-6 cytokine. We verified by quantitative RT-PCR analysis that, after transient transfection, the cells expressed high levels of *IL23R* and that the transfection did not affect *Gp130* expression levels (Fig 4A). We used these cells as a model system to monitor for IL-39-induced STAT3 pathway activity. Whereas stimulation of cells with the positive control cytokine IL-6 potently induced SEAP secretion by the HEK-Blue^TM^ IL-6 cells, addition of the IL-39 IgG1-Fc fusion protein or the concentrated supernatants containing IL-39 did not result in any enhanced SEAP secretion above that observed with non-stimulated cells (Fig 4B). In summary, we were unable to assign a functional role of IL-39 as evidenced by the lack of upregulation of reported IL-39-controlled targets and by the inability to activate the STAT3 pathway. In a final effort to probe for a biological function of IL-39, we next investigated whether the p19/EBI3 heterodimer could serve as an IL-23 receptor antagonist leading to inhibition of IL-23-induced activation of STAT3. In order to obtain an IL-23-dependent readout, the IL-23 responsive HEK293 cytokine reporter cell line stably expressing a functional IL-23 receptor linked to a STAT3-inducible SEAP reporter gene (HEK-Blue^TM^ IL-23) was utilized. Stimulation of these cells with human recombinant IL-23 resulted in STAT3 activation in a concentration-dependent manner as reflected in increased SEAP reporter gene activity (Fig 4C). Compared to IL-23, sole addition of the IL-39 IgG1-Fc fusion or the IgG1-Fc control protein to the cells did not lead to any STAT3 pathway activation (Fig 4C). When the cells were co-incubated with IL-23 (20 pM) plus various concentrations of IL-39 IgG1-Fc fusion or the IgG1-Fc control proteins IL-23-dependent STAT3 activity was not altered, even when IL-39 was used at 10-fold molar excess (Fig 4D). Stimulation of cells with 2 pM of IL-23 induced a 2.5-fold increase in STAT3 activity over background levels (Fig 4E). Various concentrations of IL-39 (up to 200 pM) were co-incubated with 2 pM of IL-23. Even at this 100-fold molar excess, we consistently found that IL-39 had no effect on IL-23-induced STAT3 activity (Fig 4E), reinforcing the idea that IL-39 does not interfere with IL-23/IL23 receptor-mediated signaling.

## Discussion

IL-39 is the most recently discovered member of the IL-6/IL-12 cytokine family and was found to be expressed as a secreted p19/EBI3 heterodimer by activated murine B cells [11]. The functional property of IL-39 is unknown, but there is evidence for a pro-inflammatory role of this cytokine. In particular, IL-39 has been implicated in the pathogenesis of murine lupus because shRNA-mediated knockdown of p19/EBI3 in B cells followed by adoptive cell transfer suppressed lupus-like disease [11]. Furthermore, administration of a polyclonal IL-39-specific antibody into mice reduced the severity of lupus symptoms [13]. Based on these findings, IL-39 may represent a target for the treatment of inflammatory diseases especially systemic lupus erythematosus. In the human system, primary keratinocytes respond to TLR3 stimulation by selectively upregulating *IL-23p19* and *EBI3* gene expression, without modifying other IL-12 family members [14]. It was postulated that IL-39 may have rather a protective role in human keratinocytes and could contribute to wound healing by dampening inflammatory responses. Furthermore, human TIGK gingival epithelial cells responded to IL-36 cytokine stimulation by upregulating *p19* and *EBI3* expression [19]. However, in these studies, the authors did not show IL-39 protein expression, but focused rather on gene expression. More research is necessary to clarify whether IL-39 is expressed and whether it has a biological function in the human system. This is particularly important based on the impressive clinical efficacy of antibodies neutralizing IL-23 in psoriasis. Theoretically, IL-39 can contribute to the pathogenesis of psoriasis and an anti-IL-23 antibody targeting the p19 subunit likely blocks the activity of IL-39 cytokine as well.

In this study, we addressed whether IL-39 is expressed by human cells and has an immunoregulatory function. It is unclear whether the human p19/EBI3 heterodimeric complex can be secreted. In one publication, intracellular association between the p19/EBI3 subunits in transfected HEK293 cells could be demonstrated by proximity ligation assays or by co-immunoprecipitation studies of the cell lysates [14]. However, this report did not address whether IL-39 could be secreted. Another study employing various biochemical techniques using conditioned media originating from p19/EBI3 transfected HEK293T cells failed to detect secretion of the p19/EBI3 heterodimer, despite providing evidence that the individual protein subunits were expressed in the cell lysates [15]. In our study, we performed similar experiments using either tagged or untagged versions of the subunits and in both cases, we were able to identify secreted IL-39 cytokine in supernatants from transfected HEK293FT cells by employing co-immunoprecipitation or ELISA techniques. We speculate that the reasons for the discrepancy between our study and the study by Detry *et al*. [15] are that different constructs with different tags were used and different techniques were applied. In the publication by Detry *et al*., it was not addressed whether the heterodimer was formed intracellularly which makes it difficult to interpret the different outcomes between the studies.

A surprising finding from our p19 immunoprecipiation studies was that we consistently observed that the p19 subunit was detected as a secreted protein in supernatants originating from HEK293FT cells transfected with the p19 plasmid alone (Fig 1C). While it was shown in the literature that overexpressed murine p19 can be secreted as a standalone protein, the human p19 subunit could only be detected in the conditioned medium if it was complexed with IL-23p40 [17]. However, recent papers underscore the possibility that the IL-23A-p19 subunit can be secreted as a standalone protein as shown that activated neutrophils and monocytes not only secrete the IL-23 p19/p40 heterodimer in cell supernatants, but also the IL-23A-p19 subunit which was not complexed with EBI3 [20]. In addition, a recent publication described that epthelial cancer cells could secrete the p19 subunit without being complexed to p40 beta subunit [21]. A similar situation may be applicable in our cellular system, especially under conditions where the cells highly overexpress the p19 protein after transfection. Alternatively and mutually not exclusive, it may be that the secreted p19 subunit detected in the supernatants was in a complex with endogenous p40 and was thus secreted as the IL-23 heterodimer. Using a commercially available, sensitive IL-23 ELISA, we could show that HEK293FT cells were indeed able to secrete low levels of IL-23 when they were transfected with the p19 plasmid. Attempts to immunoprecipitate the p40 subunit and to detect p40 by ELISA failed because appropriate and sensitive p40 antibodies for immunoprecipitation and for ELISA have not been available.

We next attempted to identify the cellular sources for IL-39 and based on published data, B cells and keratinocytes were chosen. We could confirm that the *p19* and *EBI3* mRNAs were expressed in LPS-activated human B cells or poly IC-stimulated HaCaT keratinocytes. Because M1 polarized macrophages have pro-inflammatory properties and are able to produce IL-23, we used polarized human macrophages and could show that *p19* and *EBI3* mRNAs were upregulated especially after M1 polarization of macrophages. However, IL-39 cytokine expression or secretion by any of these cells was not detectable when the culture supernatants were subjected to ELISAs. LPS-activated murine B cells were identified as the main cellular producers of IL-39 as measured by intracellular FACS, ELISA and co-immunoprecipitation analyses [11]. In that study, more than 71% of the activated B cells co-expressed p19 and EBI3 as assessed by intracellular FACS analysis. In our experiments, it is possible that IL-39 was formed at the intracellular level, but failed to get secreted by activated B cells. Intracellular FACS analysis of LPS-activated B cells clearly showed no co-expression of the p19 and EBI3 proteins in B cells (Fig 2D), indicating a lack of protein expression rather than a secretion defect. These results suggest that there is a disconnect between gene expression and translation of endogenous IL-39 protein and we speculate that IL-39 secretion is confined to murine cells and is not present or only at low levels in human B cells. We cannot rule out that our failure to detect IL-39 by ELISA could be due to the lack of sensitivity to detect IL-39, indicating the need for sensitive reagents specific for this novel IL-39 cytokine. It was reported that B cells from lupus-prone mice co-expressed the IL-39 subunits at the intracellular level and that IL-39 is associated with murine lupus immunopathogenicity [11]. In this respect, it would be interesting to extend our study and analyze IL-39 cytokine expression in samples originating from human lupus patients.

We continued our studies and aimed to elucidate the biological function of IL-39 on human cells. We utilized a commercially available disulfide-linked IL-39 chimeric protein or we used the conditioned supernatants to stimulate human neutrophils, PBMCs and IL-39 receptor positive HEK-STAT3 reporter cells. Irrespective of the cell type used, neither the IL-39 chimeric protein nor the concentrated supernatants triggered any response in our target cells. Some of the members of the IL-12 family cytokines can function autonomously as monomers or homodimers and could affect the function of their heterodimeric cytokine counterpart. In the mouse system, secreted IL-12 p40 homodimers can exist and bind to the IL-12 receptor exerting biological activities, such as antagonizing IL-12 signaling or promoting cell-mediated immune responses [22–24]. In order to assess whether IL-39 could interfere with IL-23 stimulation we obtained an IL-23-dependent cellular system and determined the impact of IL-39 addition on IL-23-induced signaling. Concomitant incubation of cells with IL-39 and IL-23 did not affect the IL-23-dependent cellular response, excluding that IL-39 could act as an IL-23 receptor antagonist. Throughout this study, we used an IL-39 IgG1-Fc fusion protein. The conformation of this protein may not reflect the real structure of IL-39, but it was reported that this protein potently stimulated mouse splenocytes resulting in increased IFNγ secretion. However, we could not confirm the reported activity using human PBMCs. A recent report by Bridgewood *et al*. used as well the same protein to stimulate human PBMCs and their results are congruent with ours [25]. This study failed to show any IL-39-induced cytokine production and STAT3 phosphorylation after exposure of cells to the IL-39 recombinant protein. No function could be assigned to IL-39, it was concluded by the authors that IL-39 remains a `theoretical cytokine`in man. We were also unable to detect any IL-39 cytokine production and to assign any biological activity to IL-39 in the human system and we speculate that IL-39 may be immunoregulatory in mice only. In our study, we based our choice of cells that could potentially produce IL-39 or respond to IL-39 on published work that was done in the mouse system. Hence, it is possible, unlike in mice, that human IL-39 is produced by other cells or that it targets different cells than published. More research is definitively needed to further elucidate the biological properties of IL-39 in human.

## Supporting information

S1 Fig IL-23p19 and EBI3 proteins interact and form a stable, secreted heterodimeric complex. (XLSX)

S2 Fig Gene expression, but no protein expression of the IL-39 subunits from human immune cells and keratinocytes. (XLSX)

S3 Fig Human IL-39 does not activate immune cells (neutrophils and T cells). (XLSX)

S4 Fig Human IL-39 does not a activate STAT3 in reporter assay and interfere with IL-23-dependent signaling in a reporter cell line. (XLSX)

S1 Raw images (TIF)
